# Transcriptome analysis of human heart failure reveals dysregulated cell adhesion in dilated cardiomyopathy and activated immune pathways in ischemic heart failure

**DOI:** 10.1186/s12864-018-5213-9

**Published:** 2018-11-12

**Authors:** Mary E. Sweet, Andrea Cocciolo, Dobromir Slavov, Kenneth L. Jones, Joseph R. Sweet, Sharon L. Graw, T. Brett Reece, Amrut V. Ambardekar, Michael R. Bristow, Luisa Mestroni, Matthew R. G. Taylor

**Affiliations:** 1 Human Medical Genetics and Genomics, University of Colorado, Aurora, CO USA; 2 Cardiovascular Institute and Adult Medical Genetics Program, University of Colorado, Aurora, CO USA; 3 Department of Pediatrics, Section of Hematology, Oncology, and Bone Marrow Transplant, University of Colorado, Aurora, CO USA; 4 Department of Statistics, E. & J. Gallo, Modesto, CA USA; 5 Department of Cardiothoracic Surgery, University of Colorado Hospital, Aurora, CO USA; 6 Division of Cardiology, Department of Medicine, University of Colorado, Aurora, CO USA

**Keywords:** RNA-seq, Transcriptome, Gene expression, Cardiomyopathy, Heart failure

## Abstract

**Background:**

Current heart failure (HF) treatment is based on targeting symptoms and left ventricle dysfunction severity, relying on a common HF pathway paradigm to justify common treatments for HF patients. This common strategy may belie an incomplete understanding of heterogeneous underlying mechanisms and could be a barrier to more precise treatments. We hypothesized we could use RNA-sequencing (RNA-seq) in human heart tissue to delineate HF etiology-specific gene expression signatures.

**Results:**

RNA-seq from 64 human left ventricular samples: 37 dilated (DCM), 13 ischemic (ICM), and 14 non-failing (NF). Using a multi-analytic approach including covariate adjustment for age and sex, differentially expressed genes (DEGs) were identified characterizing HF and disease-specific expression. Pathway analysis investigated enrichment for biologically relevant pathways and functions. DCM vs NF and ICM vs NF had shared HF-DEGs that were enriched for the fetal gene program and mitochondrial dysfunction. DCM-specific DEGs were enriched for cell-cell and cell-matrix adhesion pathways. ICM-specific DEGs were enriched for cytoskeletal and immune pathway activation. Using the ICM and DCM DEG signatures from our data we were able to correctly classify the phenotypes of 24/31 ICM and 32/36 DCM samples from publicly available replication datasets.

**Conclusions:**

Our results demonstrate the commonality of mitochondrial dysfunction in end-stage HF but more importantly reveal key etiology-specific signatures. Dysfunctional cell-cell and cell-matrix adhesion signatures typified DCM whereas signals related to immune and fibrotic responses were seen in ICM. These findings suggest that transcriptome signatures may distinguish end-stage heart failure, shedding light on underlying biological differences between ICM and DCM.

**Electronic supplementary material:**

The online version of this article (10.1186/s12864-018-5213-9) contains supplementary material, which is available to authorized users.

## Background

Heart failure (HF) affects an estimated 6.5 million adult Americans [1]. Although survival rates have improved by 10% between 1979 and 2000, the current 5-year mortality rate is still ~ 50% [1, 2]. A long-standing paradigm is that later-stage heart failure with reduced ejection fraction (HFrEF) evolves via a “final common pathway” despite having diverse etiologies and genetic contributions [3, 4]. Clinical trial results and current guidelines for HFrEF management reflect this viewpoint and direct therapy based largely on the degree of left ventricular dysfunction, assessed by ejection fraction, and clinical severity using the New York Heart Association (NYHA) classification [5]. Despite HFrEF due to ischemic cardiomyopathy (ICM) having a worse prognosis than dilated cardiomyopathy (DCM) [6, 7], current therapies are relatively indifferent to disease etiology, potentially reflecting an incomplete understanding of the heterogeneous biological mechanisms contributing to HFrEF. Animal and cell-based HF models have provided key insights into general HF biology, but have rendered a more limited contribution into subtypes of HF and into the substantial variation that is present in patient populations as compared to homogenous strains in animal and cell-based models. An improved understanding of underlying human HF biology could provide insight into diverse mechanisms and pave the way for new precision medicine strategies. We employed a global transcriptomics approach to uncover biological pathways that characterize human HFrEF of general ICM or DCM etiology.

Prior microarray studies suggested distinct gene expression signatures between HF etiologies [8–13]; but others failed to find distinctions [14, 15]. Direct RNA-sequencing (RNA-seq) provides superior quantification of transcripts compared to microarrays and has been used to identify expression signatures between HF and non-failing (NF) hearts [16], novel transcriptional regulators and perturbed miRNA networks in ICM or DCM [17, 18], pre- and post-LVAD transcriptomes [19], common HF genes in pediatric cardiomyopathy [20], and splicing, eQTL, and allelic expression in DCM [21]. Less progress has been made in refining differential expression between different adult HFrEF etiologies, particularly in human tissue models where access is often limited and sample sizes are small. Additionally, prior studies focused on comparing end-stage diseased hearts and NF hearts, which allows for little intra-disease resolution. Overcoming the challenges of accessing human tissues, the previous largest RNA-seq study of HF etiologies consisted of 13 ICM, 13 DCM, and 10 NF, but the analyses focused on cytoskeletal and transport genes as translational targets in HF rather than distinguishing disease-specific pathways within HF [22–24].

**Fig. 1:**
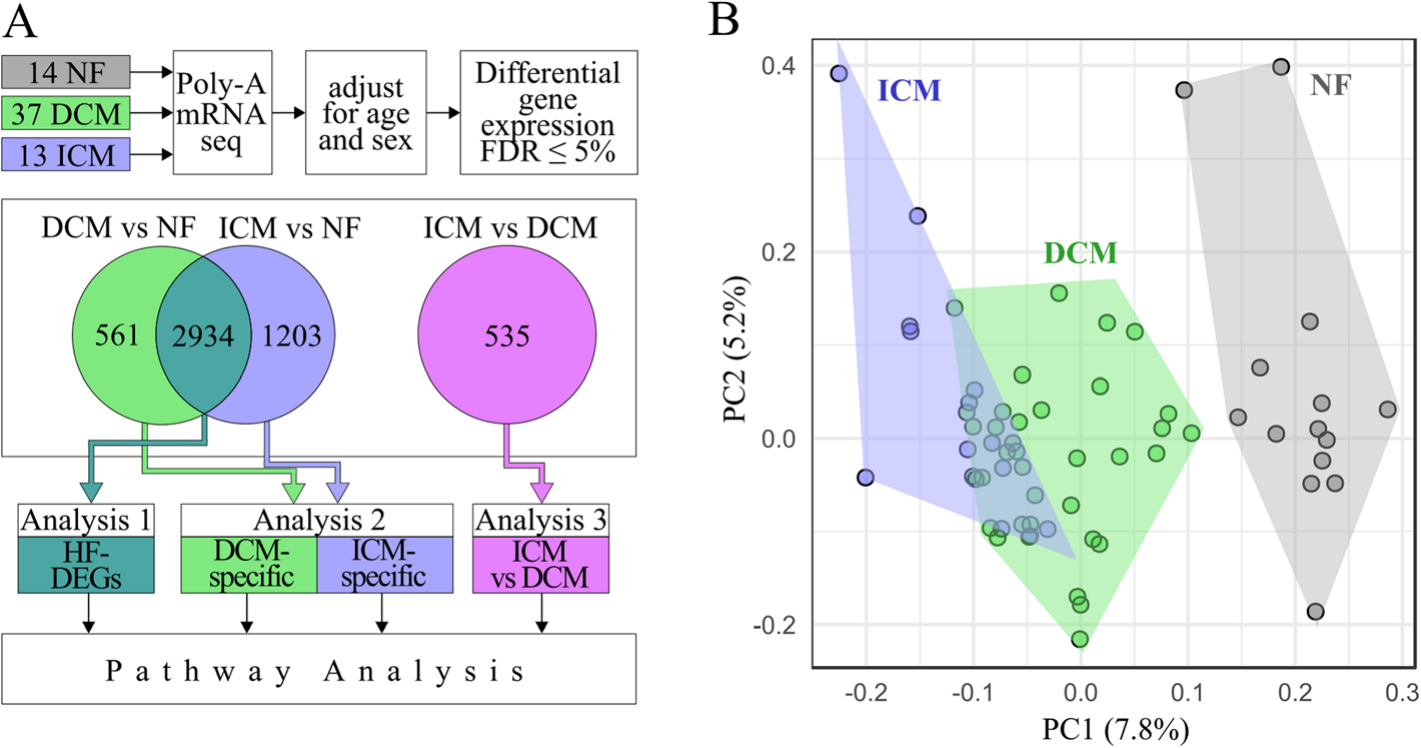
Schematic of RNA-seq analyses. **a** mRNA from 64 human hearts was extracted, sequenced, and adjusted for covariates. By comparing DEGs at an FDR of 5%, three pathway analyses were conducted. Analysis 1 considered all shared DEGs between DCM vs NF and ICM vs NF as HF-DEGs (green-blue). Analysis 2 considered non-overlapping DEGs as DCM-specific (green) or ICM-specific (blue). Analysis 3 directly compares diseases (pink). **b** Principal component analysis of all three cohorts, ICM (blue), DCM (green), and NF (grey). On the first two principal components, each of the three groups clusters together with overlap between ICM and DCM. ICM clusters further away from NF than DCM

We used RNA-seq in human left ventricles to resolve distinct etiologies within HF, specifically between ICM and DCM. Our hypothesis was that etiology-specific transcriptome signatures exist and can distinguish disease-specific HF mechanisms. Our analysis gives insight into a potentially etiology-specific pathogenesis of HFrEF, providing evidence that a single final common pathway may not fully characterize HF and that HF can be sub-classified into etiology-specific expression signatures. We performed RNA-seq on 64 explanted human hearts, using a multi-analytic approach to demonstrate there are common HF pathways as well as disease-specific signatures in DCM and ICM (Fig. 1a). Our results support shared and unique mechanisms in heart failure etiologies that contribute to HFrEF.

## Results

### Clinical characteristics of patients

Sixty-four hearts were investigated: 37 from DCM patients, 13 from ICM patients, and 14 NF (Additional file 1: Table S1). Table 1 summarizes clinical characteristics between the patient groups. The ICM cohort had a significantly greater proportion of patients taking statins (*p* < 0.0001) and having coronary artery disease (*p* < 0.0001), hyperlipidemia (*p* = 0.005) and diabetes mellitus (*p* = 0.004).

**Table 1:** Clinical characteristics of DCM and ICM cohorts

Characteristics	DCM (*n* = 37)	ICM (*n* = 13)	*p*-value
Male sex, *n* (%)	30 (81)	10 (77)	0.71
Age at transplant	49 ± 13	56 ± 4	0.10
Race			
Caucasian, *n* (%)	31 (84)	13 (100)	0.32
Black/African American, *n* (%)	3(8)	0 (0)	0.56
unknown, *n* (%)	3(8)	0 (0)	0.56
Ethnicity			
Not Hispanic or Latino, *n* (%)	26 (70)	7 (54)	0.32
Hispanic or Latino, *n* (%)	5 (14)	1 (8)	1.00
unknown, *n* (%)	6 (16)	4 (31)	0.42
NYHA	3.3 ± 0.6	3.3 ± 1	0.67
^a^LVEF (%)	18 ± 8	13 ± 5	0.09
Comorbidities			
Coronary artery disease, *n* (%)	4 (11)	13 (100)	< 0.0001
Diabetes mellitus, *n* (%)	6 (16)	8 (62)	0.004
Hyperlipidemia, *n* (%)	8 (22)	9 (69)	0.005
^a^History of smoking, *n* (%)	17 (49)	8 (67)	0.33
Hypertension, *n* (%)	16 (43)	8 (62)	0.34
^a^BMI ≥ 30, *n* (%)	5 (16)	2(22)	0.64
Medications			
Inotropes, *n* (%)	11 (30)	3 (23)	0.73
Statins, *n* (%)	10 (27)	12 (92)	< 0.0001
Antiarrhythmics, *n* (%)	32 (86)	12 (92)	1.00
Amiodarone, *n* (%)	11 (30)	3 (23)	0.73
Aspirin, *n* (%)	8 (62)	15 (41)	0.22
Beta Blockers, *n* (%)	20 (54)	8 (62)	0.75
ACE inhibitor, *n* (%)	17 (46)	8 (62)	0.52
Device Therapy			
ICD, *n* (%)	32 (86)	8 (62)	0.10
LVAD/BiVAD, *n* (%)	16 (43)	4 (31)	0.52

^a^Unknown for some patients. Plus-minus values are means ± one SD. *P*-values determined by Mann-Whitney U Test or Fisher’s Exact Test (at significance levels of 0.05, 2-tailed hypothesis) where appropriate. *ICD* implantable cardioverter defibrillator, *LVEF* left ventricular ejection fraction, *LVAD/BiVAD* left/biventricular assist device, *NYHA* New York Heart Association

### Principal components of the cohorts

To investigate gene expression differences between HFrEF etiologies, we performed single replicate poly-A RNA-seq on left ventricular tissue samples (Fig. 1a, Additional file 2: Table S2). We used principal component analysis to broadly understand gene expression relationships between cohorts and visualize sample clustering for the most variably expressed genes (Fig. 1b). Using the first two components, the samples cluster distinctly between disease and NF and by disease with some overlap. ICM samples cluster further away from NF than DCM.

### Random sample permutation

To test the strength of our disease classifications, we conducted a random sampling analysis. We show that our classifications achieve the highest number of DEGs of any random classifications and are highly significant within a 99.99% confidence interval. In DCM vs NF 96.4% of combinations had five or less DEGs, and the maximum combination had 1105 DEGs (compared to the observed 3649: M = 8.50, SD = 76.03, *p* < 2.2e^− 16^; Additional file 3: Figure S1a). In ICM vs NF, 98.1% of combinations had five or less DEGs, and the maximum combination had 1940 DEGs (compared to the observed 4150: M = 9.59, SD = 106.29, *p* < 2.2e^− 16^; Additional file 3: Figure S1b). In ICM vs DCM, 96.8% of combinations had five or less DEGs, with the maximum combination having 560 DEGs (compared to the observed 874: M = 5.51, SD = 42.63, *p* = 4e^− 12^; Additional file 3: Figure S1c). No combination produced as many DEGs as our NF and disease cohorts, suggesting our original clinical classifications were rigorous.

### Multiple linear regression to adjust for covariates

**Fig. 2:**
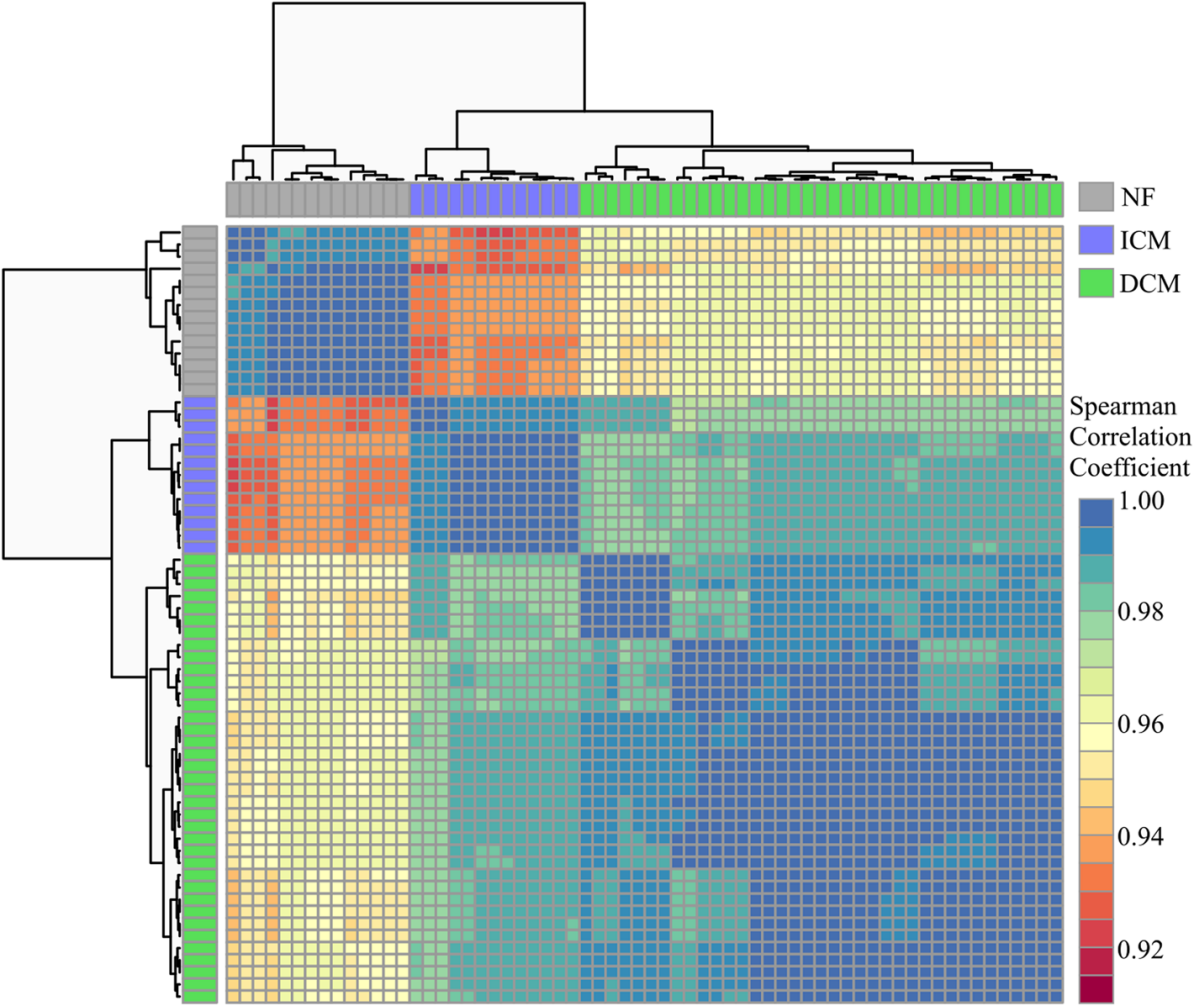
Correlation matrix between samples. The heatmap matrix shows the Spearman correlation coefficient between samples for all expressed genes following adjustment. Samples cluster by phenotype. Cooler colors (blues, greens) represent relationships between samples that are most similar; warmer colors (reds, oranges) represent samples that are more dissimilar with lower coefficients

Because age and sex are known to contribute to heart failure risk, we used multiple linear regression to adjust the gene expression for these confounding effects. Spearman correlation between the samples demonstrated that following covariate adjustment, the samples clustered into three distinct phenotypic groups with the NF and ICM groups being the most dissimilar (Fig. 2). Before adjustment, DCM vs NF had 3649 DEGs; after applying the model to adjust the expression for differences in age and sex, there were 3495 DEGs. A majority (3419; 98%) were significant before the model. ICM vs NF had 4150 DEGs and 4137 after the model was applied. A majority (3808; 92%) were significant before the model. ICM vs DCM had 874 DEGs, 535 DEGs after the model was applied, and 499 (93%) were significant before the model. Unadjusted DEGs are included in Additional file 4: Table S3, and the adjusted gene expression values were used for the remaining analyses.

### Analysis 1: HF-DEGs

**Fig. 3:**
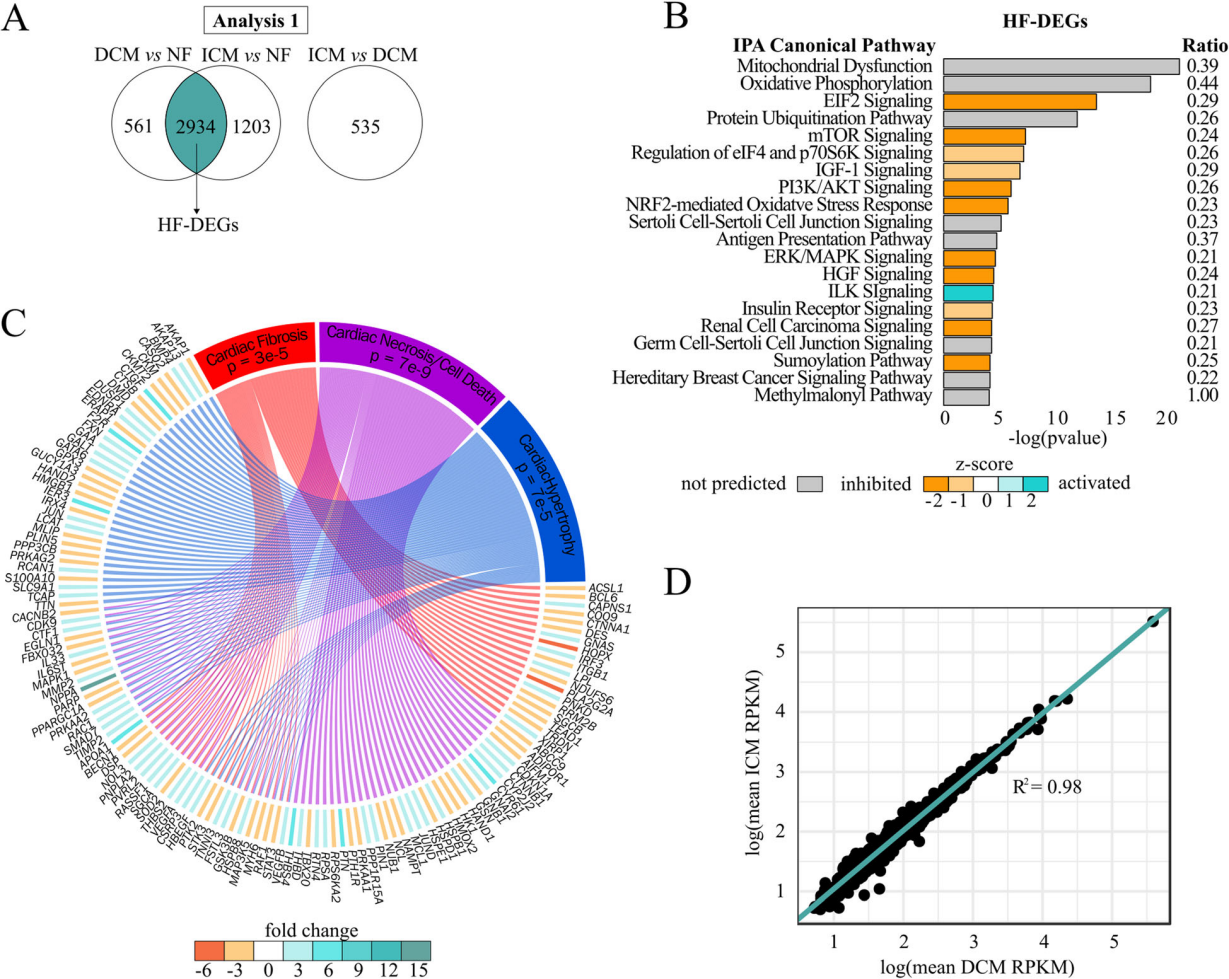
Pathway analysis in HF-DEGs. **a** Venn diagram of DCM vs NF and ICM vs NF DEGs highlighting 2934 overlapping genes used in this analysis. **b** Top 20 enriched pathways. Bars are filled according to z-score: teal indicates higher (activated), orange indicates lower (inhibited). Pathways without a z-score are grey, and pathways with a z-score of zero are white. The ratio of the number of enriched genes to the number of total genes in the pathway is listed on the right side. **c** Circos plot of enriched biofunctions and their corresponding DEGs according to IPA. DEGs are colored by mean fold change from DCM or ICM vs NF. **d** Scatter plot of mean RPKM values of DCM against ICM logarithmically (R^2^ = 0.98) for the 2934 HF-DEGs

There are 2934 HF-DEGs (1472 upregulated, 1462 downregulated; Fig. 3a, Additional file 5: Table S4). Many of these genes agree with previous HF gene expression literature, including decreased *MYH6* (fold change = DCM, − 1.5; ICM, − 2.0) expression and increased *NPPA* (fold change = DCM, 18.1; ICM, 11.2) and *NPPB* (fold change = DCM, 15.0; ICM, 22.4) expression (Additional file 5: Table S4) [25, 26]. The four most significant pathways are Mitochondrial Dysfunction, Oxidative Phosphorylation, EIF2 Signaling, and Protein Ubiquitination Pathway (Fig. 3b, Additional file 6: Table S5). Toxicity annotation in IPA revealed significant enrichment of well-characterized HF pathologies including cardiac fibrosis, hypertrophy, and necrosis/cell death (Additional file 7: Table S6). The genes involved in these pathologies that are dysregulated in the HF-DEGs are illustrated in Fig. 3c.

The fold change direction for HF-DEGs was the same in both diseases for all genes. When plotting the average RPKM values for one disease against the other logarithmically, R^2^ = 0.98 (Fig. 3d), indicating correlation of the relative magnitude of gene expression. This suggests these genes represent an expression pattern common to a failing heart irrespective of disease phenotype.

### Analysis 2: disease-specific

#### Identifying disease-specific DEGs

**Fig. 4:**
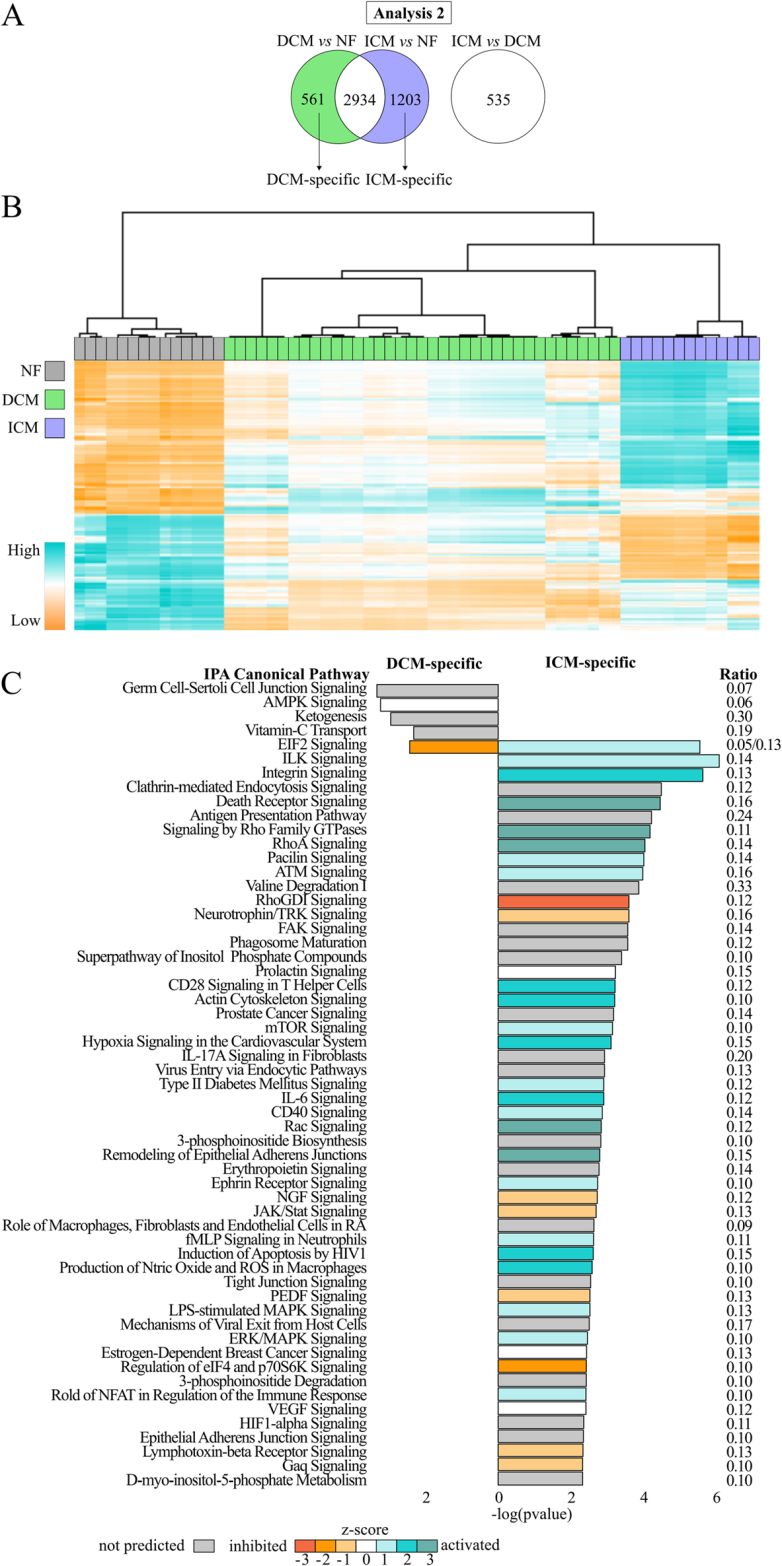
Pathway analysis in disease-specific DEGs. **a** Venn diagram of DCM vs NF and ICM vs NF highlighting 561 DCM-specific (green) and 1203 ICM-specific (blue) DEGs in this analysis. **b** Unsupervised clustering heatmap of DCM- and ICM-specific DEGs. Samples cluster according to etiology. **c** Enriched pathways (*p* ≤ 0.005). DCM-specific (left) and ICM-specific (right)

By removing the HF-DEGs from each comparison, DCM vs NF had 561 DCM-specific DEGs (202 upregulated, 359 downregulated) and ICM vs NF had 1203 ICM-specific DEGs (814 upregulated, 389 downregulated; Fig. 4a, Additional file 5: Table S4). To validate the disease specificity of these 561 and 1203 gene profiles, we performed hierarchical clustering of the combined genes for all samples to visualize gene expression clustering. The samples segregate into three large distinct clusters by NF, DCM, and ICM (Fig. 4b). The NF and ICM samples cluster relatively homogenously compared to DCM, which clusters into smaller heterogeneous groups.

#### Disease-specific DEG validation

To independently validate the disease specificity of the DCM- and ICM-gene profiles, we used four previously published datasets that are publicly available in the NCBI GEO database: microarray data from GSE1145 and GSE1869 and RNA-seq data from GSE55296 and GSE46224 [10, 18, 19, 23, 24]. From GSE1869 and GSE46224, only post-transplant data was compared. Samples from GSE1869 and GSE46224 included ICM (*n* = 7 and *n* = 8, respectively) and non-ischemic cardiomyopathy (NICM) (*n* = 8 for both GSE1869 and GSE46244), and we assumed that clinical NICM was largely equivalent to DCM. We extracted expression values for the 561 and 1203 disease-specific genes from each dataset. Using the same hierarchical clustering methods, we demonstrated that this disease-specific expression profile was able to accurately segregate 10/13 DCM and 9/13 ICM from GSE55296 (Additional file 8: Figure S2a), 8/8 NICM and 5/7 ICM from GSE1869 (Additional file 8: Figure S2b), 14/15 DCM and 10/11 ICM from GSE1145 (Additional file 8: Figure S2c), but was not sufficient to accurately segregate samples from GSE46224 (Additional file 8: Figure S2d).

#### Pathway analysis for DCM-specific DEGs

**Fig. 5:**
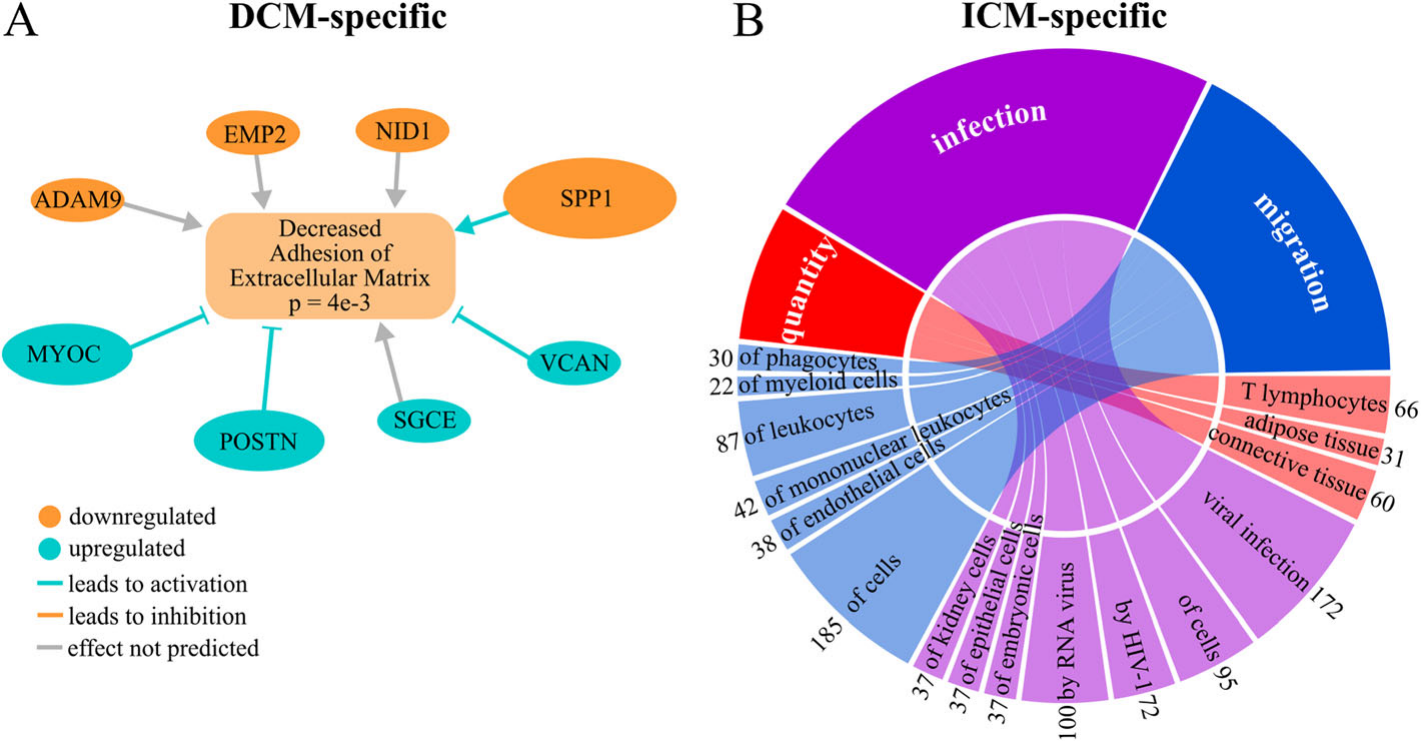
Pathway analysis in disease-specific DEGs. **a** Network of genes involved in the predicted decrease of extracellular matrix adhesion in DCM. The absolute fold change of each gene is indicated by the size of its oval. **b** Circos plot of predicted activated biofunctions in ICM for three categories: quantity, infection, and migration. Connection sizes correlate to the number of genes involved in each sub-category, which are listed on the outside of the circle

At *p* ≤ 0.05, 47 pathways were predicted to be enriched (Additional file 6: Table S5). Those with *p* ≤ 0.005 are listed on the left side of Fig. 4d. The most significantly enriched pathways are Germ Cell-Sertoli Cell Junction Signaling, implicating involvement of intercellular adhesion, and AMPK, which aids in monitoring heart energy consumption [27]. Functional annotation of DEGs revealed decreases in adhesion, cell survival, and metabolism of reactive oxygen species (Additional file 9: Table S7) The differential expression of genes involved in the extracellular matrix are predicted to decrease extracellular matrix adhesion (Fig. 5a).

#### Pathway analysis for ICM-specific DEGs

At *p* ≤ 0.05, 153 enriched pathways were predicted (Additional file 6: Table S5). Those pathways with *p* ≤ 0.005 are listed on the right side of Fig. 4d. The most enriched pathways are ILK and Integrin Signaling and the most significantly activated pathways are RhoA and Death Receptor signaling. Many enriched pathways involve the immune system, including Antigen Presentation, CD28 in T Helper Cells, IL-6, CD40, JAK/Stat, fMLP in Neutrophils, and role of NFAT in Regulation of Immune Response. There is also enrichment for activation of cytoskeletal regulation pathways: ILK, Integrin, Rho Family GTPases, RhoA, RhoGDI, Actin Cytoskeleton, Rac, and Remodeling of Epithelial Adherens Junctions. Functional annotation revealed increased infection and quantity and migration of multiple immune cells (Fig. 5b, Additional file 9: Table S7). One pathway is shared between the disease-specific comparisons: EIF2, which is predicted to be activated in ICM and inhibited in DCM.

### Analysis 3: ICM vs DCM direct transcriptome comparison

**Fig. 6:**
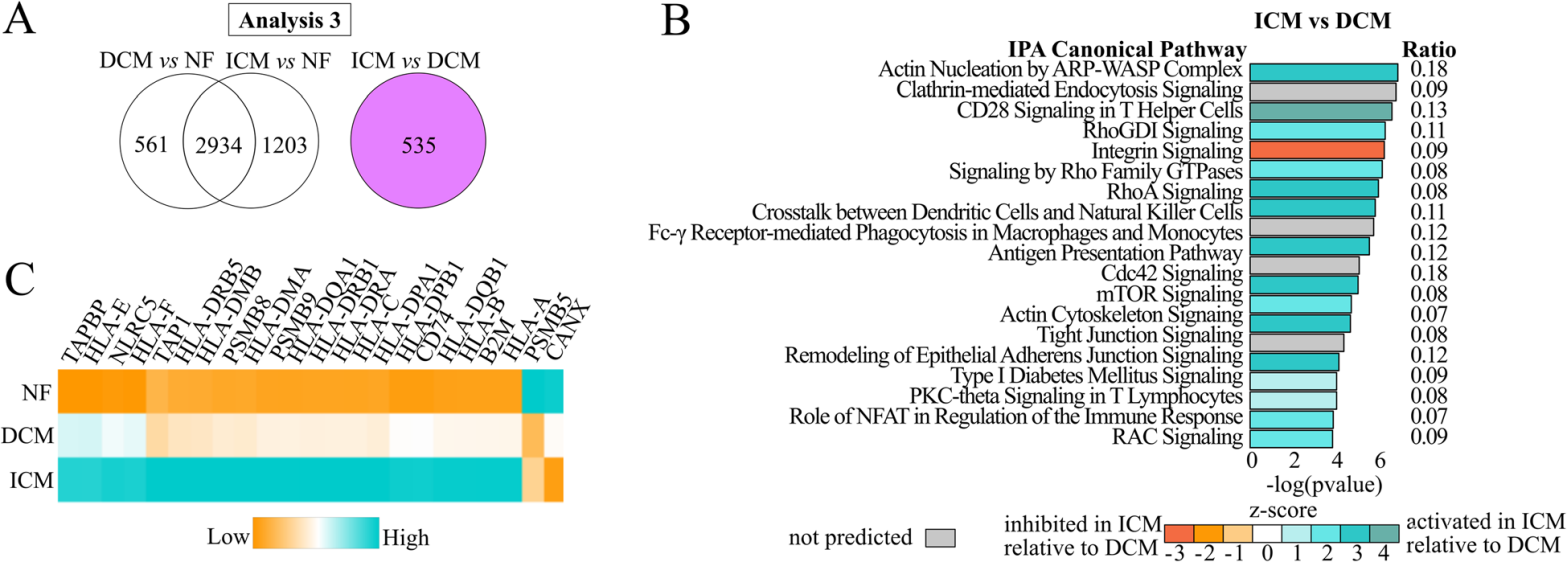
Pathways enriched in ICM vs DCM. **a** Diagram highlighting 535 DEGs from ICM vs DCM. **b** Top 20 enriched pathways. Teal indicates higher (activated) in ICM relative to DCM, and orange indicates lower (inhibited) in ICM relative to DCM. **c** Graded activation of Antigen Presentation Pathway from relative low expression in NF, moderate expression in DCM, and high expression in ICM

Lastly, we confirmed these results reflected significant differences in gene expression between the two HFrEF general etiologies by directly comparing the DCM and ICM transcriptomes. We identified 535 DEGs (Fig. 6a). 356 (67%) are upregulated in ICM relative to DCM. Using IPA, 121 pathways are significantly enriched (*p* ≤ 0.05; Additional file 6: Table S5), and Fig. 6b illustrates the 20 most significant pathways. Thirteen of these pathways (Integrin, Clathrin-mediated Endocytosis, Antigen Presentation, Rho Family GTPases, RhoA, RhoGDI, CD28 in T Helper Cells, Actin Cytoskeleton, mTOR, Rac, Remodeling of Epithelial Adherens Junctions, Tight Junction, and Role of NFAT) were enriched in the ICM-specific Analysis 2 in the same z-score directions, suggesting their significance in this comparison is due to upregulated genes in ICM rather than downregulated genes in DCM.

#### Antigen presentation pathway

The Antigen Presentation Pathway was a significantly enriched pathway in all four analyses. We investigated its gene expression more deeply. The heatmap in Fig. 6c depicts the average expression of each significant gene from the pathway in the three cohorts. It demonstrates a graded activation of Antigen Presentation Pathway genes, with relatively low NF expression, moderate DCM, and high ICM expression.

## Discussion

### There is a common HF transcriptome signature characterized by general metabolic dysfunction

Our HF-DEG analysis confirmed the hallmark fetal gene expression of HF controlled by β1-adrenergic receptor signaling [28, 29] and revealed additional critical common HF pathways supporting overall metabolic dysfunction in the failing heart. All 2934 HF-DEGs were in the same fold change direction at similar expression magnitudes for both diseases compared to NF, suggesting these genes are characteristic of a failing heart (Fig. 3d). Many of these genes also correspond to known heart failure pathologies (Fig. 3c), The top four pathways enriched in this gene set were Mitochondrial Dysfunction, Oxidative Phosphorylation, EIF2 Signaling, and Protein Ubiquitination Pathway (Fig. 3b). When comparing gene expression of HF to NF, mitochondrial pathways have commonly been disrupted, not only in microarrays of human tissue [30, 31] but also in microarray and RNA-seq of animal models of HF [32–34]. Insufficient energy production in the failing heart has long been known to contribute to left ventricular dysfunction. Oxidative phosphorylation is decreased in chronic HF 30–50% [35] and decreased mitochondrial enzyme levels have been associated with HF severity [36] and mortality [37]. Targeting mitochondrial function in HF has been recognized as having tremendous untapped potential and is currently a forefront target for novel HF therapies [38]. EIF2 signaling is required for translation, but is inhibited, suggesting decreased protein production. The Protein Ubiquitination Pathway is enriched, suggesting an increase in protein degradation due to cell death or tissue necrosis.

### Cell-cell and cell-matrix adhesion is perturbed in DCM

The DCM-specific pathway analysis showed the top enriched pathway for DCM was Germ Cell-Sertoli Cell Junction Signaling (Fig. 4d, left side). Germ cell-sertoli cell junctions in the testis are desmosome-like, comprised of many of the same proteins as cardiac desmosomes, and are essential for cell-cell adhesion and intercellular signal transduction [39]. In this pathway, genes encoding microtubule subunits, or tubulins, are downregulated: *TUBA1B* encoding an α-tubulin, *TUBB4B* encoding a β-tubulin, and *TUBG1* encoding a γ-tubulin. In NF, *TUBA1B* and *TUBB4B* are two of the three highest expressed tubulins. This agrees with a recent publication reporting that *TUBA3D* and *TUBA3E* were significantly downregulated in DCM [21]. Evidence suggests microtubules are responsible for transporting gap junction protein connexin-43 to the cell surface [40], and gap junction remodeling, including reduced expression of connexin-43 in myocytes, occurs in DCM [41, 42]. This junction signaling pathway was also confirmed as being enriched in the direct comparison between ICM vs DCM.

Increased expression of *MYOC*, *POSTN*, *SGCE*, and *VCAN* (fold changes = 2.1, 2.7, 1.2, 1.7 respectively) and decreased expression of *ADAM9*, *EMP2*, *NID1*, and *SPP1* (fold change = − 1.5, − 1.2, − 1.3, − 3.6, respectively) may contribute to decreased cell-matrix adhesion (Fig. 5a). *SGCE* encodes the epsilon component of the sarcoglycan transmembrane complex, which connects the cardiomyocyte to the extracellular matrix. *MYOC*, *POSTN*, *VCAN*, *ADAM9*, *NID1*, and *SPP1* all reside primarily in the extracellular space. In particular, *SPP1* expression plays a protective role in cardiac dilation, possibly by promoting fibroblast growth and adhesion [43]. *POSTN* encodes periostin, which is known to be highly expressed in HF caused by DCM. Overexpression of *POSTN* inhibits myocyte spreading and fibroblast adhesion, and it contributes to cardiac dysfunction [44]. Additionally, cell adhesion and cytoskeletal processes have been previously implicated in DCM [16, 18] and mutations in cytoskeletal genes are known to cause DCM [45], potentially through disrupted mechanotransduction.

### The immune system and cytoskeleton are activated in ICM

The ICM-specific pathways can be categorized into two main types, immune response and cytoskeletal regulation (Fig. 4d, right side). Involvement of these in ICM may stem from response to two stimuli, which are not mutually exclusive: the damaged, infarcted myocardium, to which the immune system responds through inflammation followed by fibrotic scar formation, and atherosclerosis, which is the buildup of cholesterol on artery walls, currently believed to be a chronic inflammation of arterial walls eliciting a similar immune response [46, 47]. Myocardial infarctions are caused by coronary artery obstruction due to atherosclerosis, buildup of cholesterol-laden plaques on artery walls and subsequent plaque rupture with thrombus formation. In both cases, inflammation from infarcted tissue or plaque buildup and rupture induces proinflammatory cytokines like IL-1ß, TNFα, CD40LG, and IL-6 [47–49], which are all predicted to be activated in our IPA regulator analysis (*p* = 0.001, z-score = 2.7; *p* = 0.001, z-score = 4.0; *p* = 9e^− 6^, z-score = 1.8; *p* = 0.007, z-score = 3.3 respectively, data not shown). The TNFα membrane receptor is also upregulated (*TNFRSF1B*, fold change = 2.2) and CD40 and IL-6 Signaling are predicted to be activated. This cytokine production stimulates cell adhesion molecules like ICAM-1 (*ICAM1*, fold change = 2.9) to translocate to endothelial cell surfaces, either at inflamed atherosclerotic plaques or arteries in proximity to damaged tissue, to recruit and interact with leukocytes [46, 47]. Leukocyte accumulation involves a controlled process of tethering to the endothelium and migrating through the endothelium to infarcted tissue. This migration requires Rho signaling activation [48, 50]. RhoA and Signaling by Rho Family GTPases are both predicted to be activated, and RhoGDI, an inhibitor of these pathways, is predicted to be inhibited. This is supported by the IPA functional annotation of DEGs (Additional file 9: Table S7), which showed increased immune cell quantity and migration, specifically lymphocytes and leukocytes (Fig. 5b). A number of previous studies have shown Rho kinase inhibitors reduce ischemia/reperfusion injury by reducing infarct size [51], apoptosis, proinflammatory cytokines, and neutrophil response [52, 53]. However, Rho signaling is also a mechanism in fibrosis, and ROCK1 haploinsufficient and knockout mice demonstrated decreased fibrosis and fibroblast differentiation following myocardial stress [54, 55]. These findings are supported by previous transcriptome studies showing ICM enrichment for extracellular matrix-receptor interaction, actin filament processes, chemotaxis, inflammatory response, and cytokine activity [16, 22].

### Validation of ICM and DCM signatures in public datasets

We utilized four previously published datasets to test the reproducibility of our ICM and DCM signatures. Using two microarray and two RNA-seq datasets, we validated our signature in three out of four available datasets by demonstrating the correct segregation of 24/31 ICM and 32/36 DCM. Our signature was not validated in GSE46224, a dataset derived from total RNA-seq of LV apex samples. Reasons for this lack of reproducibility include differences in RNA-seq approach (total versus poly-A), different tissues (LV apex versus general LV). The higher presence of diabetes mellitus (47% in GSE46244 compared to 28% in our data) or other unmeasured variables may also explain the lack of reproducibility of our findings in GSE46244 samples. Our signature was confirmed in two microarray datasets including GSE1869, which utilized samples from two distinct institutions. This demonstrates that our results are reproducible among multiple institutions and across-platforms.

### The ICM transcriptome signature is more distinct from NF than DCM

Overall, the expression profile of ICM is more extreme compared to NF than DCM. This is evident in two aspects: 1) the principal component plot (Fig. 1b) demonstrated although the gene expression of ICM and DCM is distinct from NF, ICM was more dissimilar; this is even more evident following covariate adjustment (Fig. 2). 2) in the disease-specific analysis, there were more than twice as many ICM-specific genes as DCM-specific (1203 vs 561), which demonstrated more genes characterize ICM-specific expression. This is contrary to transcriptome comparisons following LVAD support, where mRNA profiles between ICM and NICM were not distinct [19]. This difference may be due to the fact that our samples were obtained from a later stage of HF or due to our increased sample size and power to detect differences. We also note that the DCM samples show greater heterogeneity in their transcriptomes than ICM samples (Figs. 1b, 2). This heterogeneity could be attributed to differences in pathogenic mutations in the DCM samples; as genotyping was not performed in this study, the contribution of genetic heterogeneity to the transcriptome patterns remains speculative.

A specific example of this observation that ICM is more distinct from NF than DCM is related to our consistent discovery of enrichment for the Antigen Presentation Pathway, which was significant in every transcriptome comparison. Antigen presentation is an adaptive immune response where cells use Human Leukocyte Antigens (HLAs) to present endogenous or exogenous antigens for T cells to recognize. Figure 6c displays differentially regulated genes within this pathway. Graded activation from low expression in NF, moderate in DCM, and high expression in ICM suggests this pathway is important in HF but may play a larger role in ICM, where additional inflammation, injury, and tissue necrosis are involved. Transcriptome analyses from earlier microarray studies revealed immune system enrichment for antigen processing and presentation pathways and HLA gene expression [12, 56]. However, more recent RNA-seq analyses of human tissue have failed to replicate this finding [22]. Additionally, a variant residing within a non-protein-coding gene within the chromosome six major histocompatibility complex was identified via GWAS in DCM patients. Presence of this variant influenced expression of *HLA-C*, *HLA-DRB5*, *HLA-DRB1*, and *HLA-DQB1* [57], all of which were DEGs in DCM vs NF, ICM vs NF, or both.

### Statins, coronary artery disease, diabetes mellitus, and hyperlipidemia are not associated with strong gene expression changes

In addition to sex and age, we considered other possible covariates that may affect gene expression. As noted before, statins, coronary artery disease, diabetes mellitus, and hyperlipidemia are statistically significant between our cohorts. These data are not available for the majority of the NF controls, which are derived from heart transplant donors; many of whom experience surgical harvesting at external hospital sites. Thus, including these covariates in the overall model is not possible due to missing data. Considering these covariates in a DCM-ICM only analysis is problematic because they are highly correlated with disease group, leading to multicollinearity and unstable coefficient estimates. As an alternative exploration of the degree to which these may affect expression, we fit a regression model with age, sex, and each potential covariate individually to predict expression for ICM and DCM subjects. With statins and coronary artery disease, only 15 and 7 genes (FDR ≤ 0.05) were significantly associated with each respectively. No genes were significantly associated with diabetes mellitus or hyperlipidemia. These results indicated that in our data statin use, coronary artery disease, diabetes mellitus, and hyperlipidemia were not strongly associated with gene expression changes.

It is interesting to note 12/13 of our ICM patients were on statins at time of transplantation, and the most recent lipid results for most patients were within normal ranges. Total cholesterol results for the ICM cohort prior to transplantation ranged from 81 to 168 mg/d (average = 123 mg/dL), which is within normal (< 200 mg/dL). LDL cholesterol results for the ICM cohort prior to transplantation ranged from 22 to 105 mg/dL (average = 64 mg/dL), which is also within normal (< 100 mg/dL). Although this would indicate the statins worked to reduce cholesterol, statins are known to have anti-inflammatory effects. These include reducing the expression of genes encoding proinflammatory cytokines and adhesion molecules, like ICAM1, and reducing downstream signaling pathways like Rho [58], all of which are increased or activated compared to DCM and NF. Perhaps statins are working in these patients to decrease cholesterol, but not effective in their anti-inflammatory properties.

### Study limitations

Accessing human cardiac tissue specimens and developing large tissue banks are time-intensive and costly activities, circumstances that have likely limited prior analyses. In our simulation analysis > 95% of random permutation datasets identified five or fewer DEGs, strongly supporting that our findings of 3649, 4150, and 874 DEGs are unlikely due to chance (Additional file 3: Figure S1; DCM vs. NF, ICM vs. NF, and ICM vs. DCM, respectively). In order to only include only samples specifically defined as ICM and DCM to compare, our validation of signatures in previous datasets included a microarray dataset.

Our study of DCM and ICM captured mRNA of the HFrEF left ventricle in an advanced disease state, which may be distinct from mRNA at disease onset or throughout disease progression. Thus, while our data provides inferences about end-stage HF, the biology of HF initiation and progression were not directly evaluated in our data. Because clinical cardiac biopsies yield small tissue volumes, most frequently target the right ventricle, and are performed in only a subset of HF patients, studying the transcriptome in early-stage HF and in HF progression poses additional challenges beyond those of studying explanted hearts. Furthermore, while our transcriptome analyses identified statistically significant transcriptome differences between NF, DCM, and ICM, and did not take into account groups of genes that demonstrate similar directionality without statistical significance. The RNA-seq data are limited by lack of sufficient sequence depth to address differences in alternative splicing the poly-A capture limits interrogation of many noncoding RNAs. Additional studies will be necessary to identify the key epigenetic, noncoding, and protein drivers in these pathways.

The NF donor hearts we studied are distinct from normal healthy hearts as they were harvested from persons who experienced acute and ultimately fatal events that rendered them transplant donors. Although a majority of the cell volume of myocardial tissue is from cardiac myocytes, the heart is comprised of many different cell types, and although we macroscopically controlled for tissues free of overt fibrosis, our results may reflect differences in tissue composition or pathways enriched in specific cell types.

## Conclusions

**Fig. 7:**
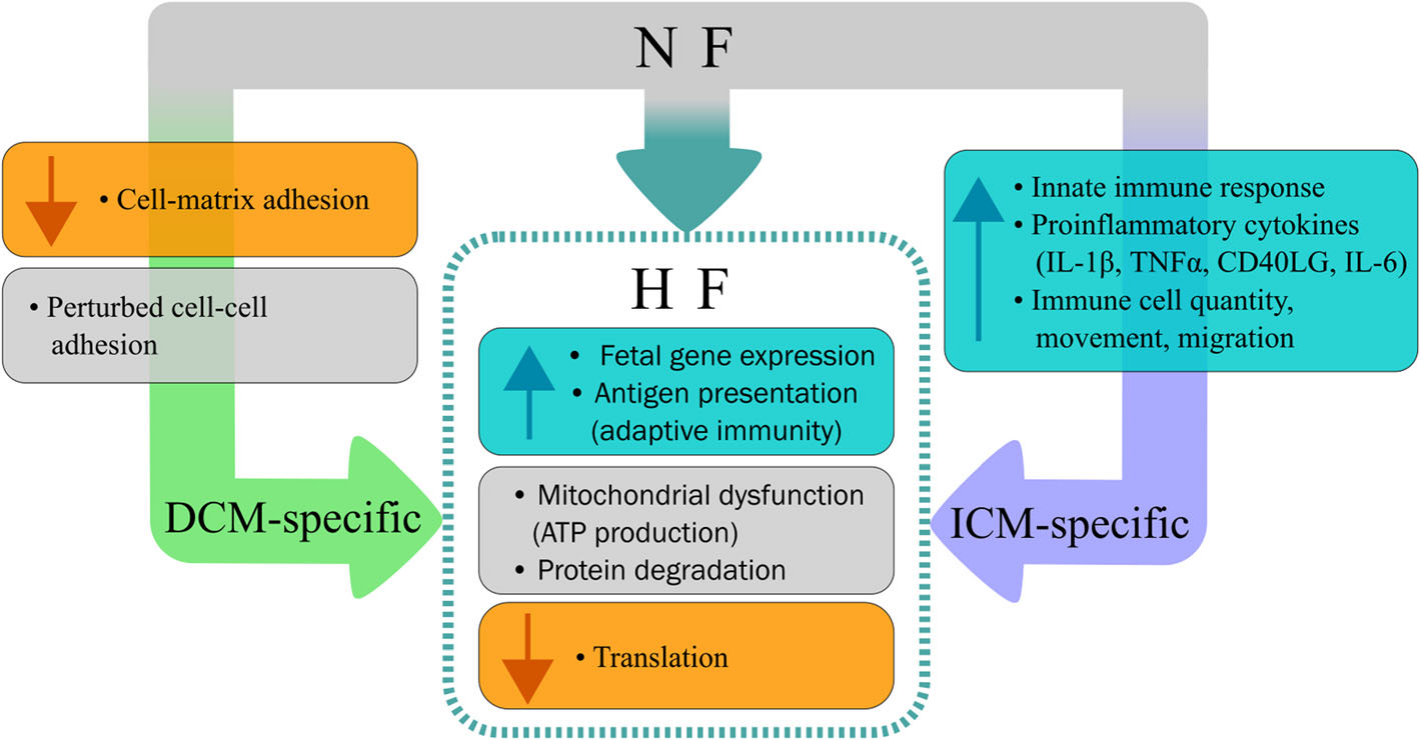
Disease-specific and shared HF pathways. Specific events can lead NF hearts towards DCM or ICM, and both diseases have common HF responses. Dysregulated cell-cell and decreased cell-matrix adhesion contributes to DCM. An activated innate immune response, activation of proinflammatory cytokines, and increases in immune cell quantity, movement, and migration are characteristic of ICM. Both DCM and ICM have responses common to HF, including reduced translation, increased fetal gene expression and antigen presentation, and dysregulated mitochondria and protein degradation

We used RNA-seq and pathway analysis in the largest cohort of human heart tissue from distinct etiologies, which is an incredibly rare and unique dataset. We demonstrate that HFrEF in left ventricles of DCM vs ICM general etiology have a common gene expression signature but also exhibit disease-specific expression signatures; our results are summarized in Fig. 7. Although the expression data does not reveal any single drivers of disease etiology, it does demonstrate that a collection of dysregulated pathways distinguishes DCM from ICM. The discovery of these key pathways in each HFrEF clinical etiology are an important step forward in heart failure genomics, and they set the stage for future functional research. These data also offer the possibility of a new taxonomic classification of HF, one of the key early steps to developing precision medicine paradigms as detailed by the National Research Council [59]. Potentially, this strategy could yield findings relevant for monitoring HF progression and designing treatments.

## Methods

### Tissue collection

Explanted failing hearts were collected from adult patients undergoing heart cardiac transplantation at the University of Colorado Hospital as part of the Division of Cardiology Cardiac Tissue Biobank under a long-standing protocol approved by the Colorado Multiple Institutional Review Board (COMIRB, protocol 01-568) where transplant-listed patients signed written consent for use of their explanted hearts for research purposes. NF left ventricular samples were obtained from organ donors whose hearts could not be placed for transplantation due to size, ABO mismatch or other factors. Family members of organ donors signed written consent for research use of explanted cardiac tissue, obtained by the local organ procurement agency. Immediately on explantation, left ventricular free wall aliquots of approximately 1 g and remote from tissue scarring or infarcted segments were immersed in liquid nitrogen, transported to the laboratory, and stored at − 80 °C.

### Patient cohorts

General clinical etiology (DCM or ICM) of patients transplanted for HFrEF was determined by medical history, based on the absence or presence of significant previously documented obstructive coronary artery disease or myocardial infarction. NF organ donor hearts were defined by no major cardiac history and a left ventricular echocardiography-based shortening fraction of ≥25%. Statistical differences between cohort clinical characteristics were calculated by either Mann-Whitney U Test or Fisher’s Exact Test, where appropriate, using a 0.05 significance level and a two-sided *p*-value.

### RNA extraction

Frozen tissue was placed in liquid nitrogen and broken up using mortar and pestle to obtain a piece approximately 2 × 2 × 2 mm in size, macroscopically free of fatty infiltration, fibrosis, and blood. Tissue was placed immediately in TRIzol reagent (Thermo Fisher Scientific, Waltham, MA) and mechanically homogenized using an IKA T25 Ultra-Turaxx homogenizer. Tissue was homogenized for approximately 60 s, or until no visible chunks of tissue remained. RNA was extracted using the *mir*Vana miRNA isolation kit (Thermo Fisher Scientific) enriched for total RNA isolation according to manufacturer’s instructions with the exception of replacing the Lysis/Binding Buffer with TRIzol. All samples were DNase treated using TURBO DNA-free Kit (Thermo Fisher Scientific). RNA was quantified at 260 nm using a NanoDrop1000 (Thermo Fisher Scientific), and RNA integrity (RIN) was measured using an Agilent 2100 Bioanalyzer with the RNA 6000 Nano Assay (Agilent, Santa Clara, CA). All samples were required to demonstrate RIN ≥ 7.0, and ranged from 7.0 to 9.3.

### RNA sequencing

PolyA transcripts were isolated from 1 μg total RNA using oligo-dT beads and the cDNA libraries were constructed using the TruSeq Stranded mRNA Library Prep Kit and protocol from Illumina (Illumina Inc., San Diego, CA). To minimize lane and batch effects, bar-coded libraries prepared from DCM, ICM, and NF samples were mixed and pooled across multiple lanes. Libraries were sequenced single-read with an Illumina HiSeq 2500 for 50 cycles at the University of Colorado Genomics and Microarray Core. The average number of reads per sample ranged from approximately 36 to 66 million with an average of approximately 48 million (Additional File 2: Table S2). Reads were filtered for quality and aligned to the GRchr37/hg19 version of the reference human genome using gSNAP [60] with an average of 95% of aligned reads Additional File 2: Table S2). Expression in terms of RPKM (reads per kilobase of transcript per million reads mapped) was derived using Cufflinks [61] and Ensembl’s GRch37.82 GTF. Due to the high proportion of cardiac mRNA reads known to map to the mitochondria [19], mitochondrial genes were removed from the GTF file for a final set of 57,974 annotations. Data have been deposited in the GEO database under GSE116250.

### Principal component analysis

Principal components were calculated in R using the svd (singular value decomposition) package and visualized in ggplot2. Principal components were used to visualize how the samples cluster for the most variably expressed genes: those genes with RPKM ≥ 5 and a difference in RPKM between disease (ICM and DCM) and NF ≥ 5 were included in the analysis.

### DEG analysis

Expressed genes were defined as genes with mean RPKM ≥ 5 in both groups. Differential expression was analyzed using Linear Model ANOVA in R. Differentially expressed genes (DEGs) were defined as genes with a difference in RPKM between groups ≥5 and a *p*-value adjusted for Benjamini-Hochberg false discovery rate (FDR) ≤ 0.05. A multiple linear regression model was used to adjust for covariates of gene expression in R. Data was transformed log2(RPKM+ 1). Disease status and sex were categorical variables and age was a continuous variable. We identified: 1) genes shared between DCM vs NF and ICM vs NF, comprising the HF transcriptome signature or “HF-DEGs,” 2) unshared, DCM- or ICM-specific genes, and 3) DEGs between DCM and ICM. We used IPA on each gene list to investigate enrichment for pathways or functions biologically relevant to each disease. IPA uses up- and down-regulation of genes to predict activation or inhibition of pathways, so gene lists were not separated by fold change.

To validate our findings, we downloaded the processed microarray probe data from GSE1145 and GSE1869, gene count data from GSE55296, and RPKM data from GSE46224 on NCBI GEO.

### Random sample permutation

For each comparison we randomly permuted groups of the same size as each cohort, without replacement, and applied our DEG pipeline, repeating 1000 times. Statistics between the observed and random sample distribution of DEGs were calculated using a one sample T-test. To graph the permutation counts logarithmically, a value of 0.1 was added to each count.

### Data visualization

Genes were clustered using Spearman rank correlation and average linkage in either Cluster 3.0 [62] or using the “cor” function with the pheatmap package in R. Clustering results were visualized in either Java TreeView [63] or pheatmap. Circos plots were created using the circlize package in R [64].

### Pathway analysis

DEGs were interpreted using Ingenuity Pathway Analysis (IPA; Qiagen, Redwood City, CA). A dataset of Ensembl gene identifiers and fold changes was uploaded for Core Analysis.

## Additional files

Additional file 1: **Table S1.** Sample table. Characteristics including sex, race, ethnicity, age, cause of death (if applicable) and RNA integrity (RIN) score are listed. (XLSX 15 kb)

Additional file 2: **Table S2.** RNAseq quality control table. Raw read counts ranged from ~ 36 to 66 million. Read counts show a 99% retention after base quality control and a 94–96% alignment rate. (XLSX 16 kb)

Additional file 3: **Figure S1.** Empirical distribution of FDR values ≤0.05 for 1000 permutations. Histogram with the number of genes in each permutation that had an FDR less than or equal to 0.05 is in logarithmic scale on the x-axis with frequency in logarithmic scale on the y axis. A value of 0.1 was added to each count to display it logarithmically. Red dotted lines indicate the observed number of DEGs in the comparison for A) DCM vs NF, B) ICM vs NF, and C) ICM vs DCM. (TIF 10663 kb)

Additional file 4: **Table S3.** DEGs for unadjusted gene expression. DEGs at FDR ≤ 0.05 in DCM vs NF and ICM vs NF. (XLSX 568 kb)

Additional File 5: **Table S4.** DEGs for adjusted gene expression. DEGs for adjusted gene expression. DEGs at FDR ≤ 0.05 in HF-DEGs, DCM-specific, ICM-specific, DCM vs ICM. (XLSX 797 kb)

Additional file 6: **Table S5.** Enriched IPA Canonical Pathways. IPA canonical pathways for *p* ≤ 0.05 for Analysis 1 (HF-DEGs), Analysis 2 (DCM-specific and ICM-specific), and Analysis 3 (DCM vs ICM). (XLSX 56 kb)

Additional file 7: **Table S6.** Enriched IPA Toxicity. IPA toxicity lists for *p* ≤ 0.05 for Analysis 1 (HF-DEGs). (XLSX 15 kb)

Additional file 8: **Figure S2.** Disease-specific DEGs clusters samples by phenotype in publicly available expression data. Expression values for the disease-specific genes identified in our analysis were extracted from publicly available datasets: A) GSE1145 (microarray), B) GSE1869 (microarray), C) GSE55296 (RNA-seq), and D) GSE46224 (RNA-seq). (TIFF 15964 kb)

Additional file 9: **Table S7.** Predicted biofunctions. IPA biofunctions predicted to be increased or decreased for Analysis 2 (DCM-specific and ICM-specific). (XLSX 34 kb)

## Abbreviations

DCM: Dilated cardiomyopathy
DEGs: Differentially expressed genes
HF: Heart failure
HFrEF: Hear failure with reduced ejection fraction
ICM: Ischemic cardiomyopathy
NF: Non-failing
NYHA: New York heart association
RNA-seq: RNA-sequencing
