# Supplementary figures and images for: Transcriptome analysis of human heart failure reveals dysregulated cell adhesion in dilated cardiomyopathy and activated immune pathways in ischemic heart failure
Source: BMC Genomics. 2018 Nov 12;19:812. doi: 10.1186/s12864-018-5213-9 (PMC6233272; doi:10.1186/s12864-018-5213-9)

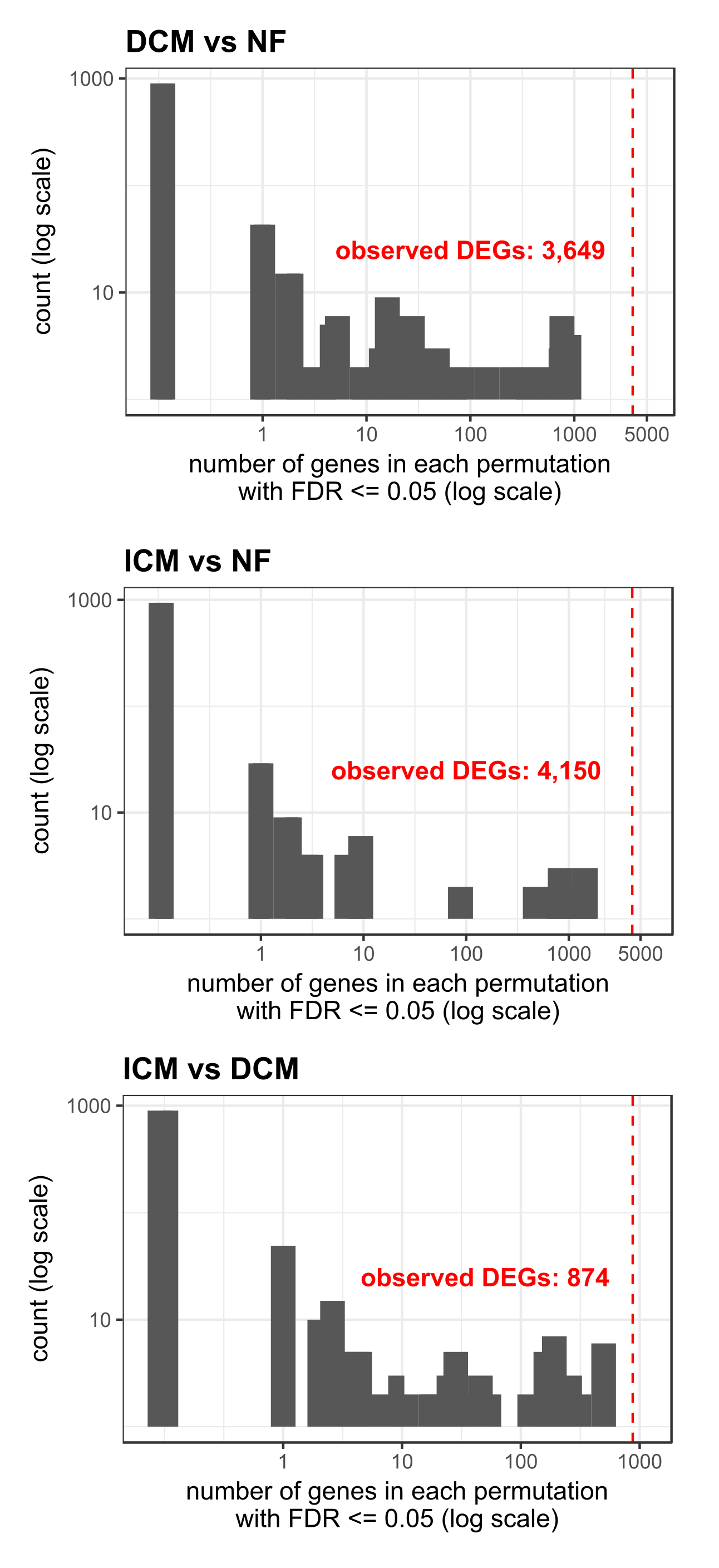

Supplement: Supplementary file 3 — Figure S1. Empirical distribution of FDR values ≤0.05 for 1000 permutations. Histogram with the number of genes in each permutation that had an FDR less than or equal to 0.05 is in logarithmic scale on the x-axis with frequency in logarithmic scale on the y axis. A value of 0.1 was added to each count to display it logarithmically. Red dotted lines indicate the observed number of DEGs in the comparison for A) DCM vs NF, B) ICM vs NF, and C) ICM vs DCM. (TIF 10663 kb) [file 12864_2018_5213_MOESM3_ESM.tif]

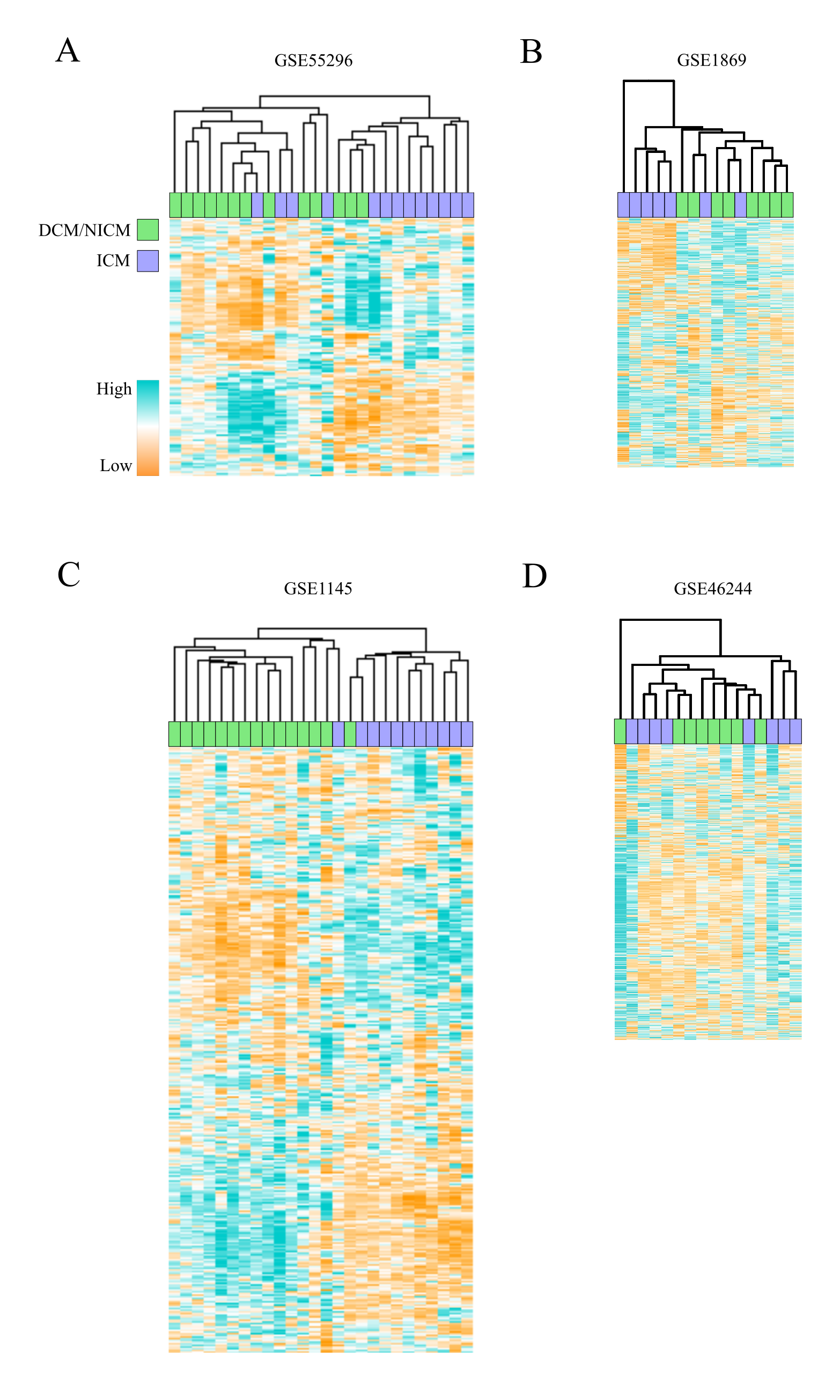

Supplement: Supplementary file 8 — Figure S2. Disease-specific DEGs clusters samples by phenotype in publicly available expression data. Expression values for the disease-specific genes identified in our analysis were extracted from publicly available datasets: A) GSE1145 (microarray), B) GSE1869 (microarray), C) GSE55296 (RNA-seq), and D) GSE46224 (RNA-seq). (TIFF 15964 kb) [file 12864_2018_5213_MOESM8_ESM.tiff]
